# Niche Specialization and Functional Overlap of Bamboo Leaf and Root Microbiota

**DOI:** 10.3389/fmicb.2020.571159

**Published:** 2020-09-18

**Authors:** Ying Zheng, Xinchun Lin

**Affiliations:** ^1^Sino-Australia Plant Cell Wall Research Centre, State Key Laboratory of Subtropical Silviculture, Zhejiang A&F University, Hangzhou, China; ^2^Zhejiang Provincial Collaborative Innovation Center for Bamboo Resources and High-Efficiency Utilization, Hangzhou, China

**Keywords:** bamboo microbiota, functional conservation, niche specialization, phylosymbiosis, plant growth promotion, structural variation

## Abstract

Leaves and roots harbor taxonomically diverse bacterial assemblages which enhance plant growth and performance by increasing nutrient supply and resistance to stress. An extensive investigation of bacterial diversity and composition between leaf and root microbiota of 15 bamboo species differing in rhizome types, lifeforms and sampling sites were conducted by high-through sequencing. The alpha diversity between leaf and root microbiota was not significantly different, whereas, their beta diversity differed remarkably. Niche specialization mainly in species from Actinobacteria was detected which prefer to colonize in roots than leaves. Community structure of leaf microbiota was highly resembled, however, the phylogeny inferred by host’s chloroplast data was incongruent with microbiota dendrogram, indicating that phylosymbiosis didn’t occur in bamboos and their associated microbiota. Large overlap in functional profiling of leaf and root-associated microbiota was found. Accordingly, we proposed that environmental conditions, structural variation and physiological differences between leaves and roots worked collaboratively for divergence of bamboo microbiota. This study confers to a robust knowledge of bamboo-microbe interaction and provides a list of bacterial lineages for investigation into specific plant–microbe interaction information of which could be used to enhance agricultural and forest productivity.

## Introduction

Plants host a diverse community of microbes known as the microbiome, which have coevolved with their hosts for millions of years (Levy et al., 2018). These phylogenetically structured microbial communities were proved to fuel the growth and fitness of host plants via nutrient supply and pathogen resistance (Muller et al., 2016). Research on various plant species, including *Arabidopsis thaliana* (eudicot) (Bai et al., 2015), rice (Zhang et al., 2019; Kim and Lee, 2020), sorghum (Emmett et al., 2017) (monocot) and *Cycas panzhihuaensis* (gymnosperm) (Zheng and Gong, 2019) has revealed that plant compartments, environmental conditions and host phylogeny are the most predominant factors influencing plant-microbial consortia (Bouffaud et al., 2014). Diverse microbes have been found in bamboo leaves, rhizomes, roots, and seeds (Shen et al., 2014; Liu et al., 2017) based on culture-dependent and independent methods (Yuan et al., 2015a; Debnath et al., 2018), some of which were endophytic phosphorus- and potassium-solubilizing bacteria (Yuan et al., 2015b; Yuan et al., 2018). The photosynthetic rate, transpiration rate, and stomatal conductance in *Phyllostachys edulis* treated with these growth-promoting bacteria were all higher than in the control groups (Yuan et al., 2018).

Plant compartments/organs serve as specific niches - environmental surroundings that affect their growth and performance - colonized by a distinctive assemblage of microbial taxa (Muller et al., 2016; Beckers et al., 2017; Zheng and Gong, 2019). The most frequently studied plant organs are roots and leaves (Muller et al., 2016; Toju et al., 2019). Bamboo leaves and roots have different microbiota: root endophytic bacteria were dominant by *Bacillus* (Han et al., 2009), while the leaf-dwelled endophytes were mainly composed by *Staphylococcus* (Xu et al., 2014). Root-associated microbiota is defined largely by soil properties and host phylogeny is responsible for the fine-tuning of community structure during the establishment of endophytic microbiota (Bulgarelli et al., 2013). The phyllosphere is considered as harsh environment with rapidly changing conditions and exposure to various stresses (Leveau, 2019; Liu et al., 2020). Although root-associated microorganisms are exposed to stress as well but they are protected from UV radiation and their environmental conditions are likely to change less frequently compared to leaf microbiome (Bulgarelli et al., 2013; Cordovez et al., 2019).

Apart from the external environmental differences, the internal structure and fundamental physiological divergence between roots and leaves also play role in shaping distinct plant–microbe partnership (Singh and Dubey, 2018). The metabolic productions, plant peptide signals, differential protease activity, and pathogen defense mechanisms are different between root and leaf compartments, resulting in the colonization by niche-specific core microorganisms (Baltrus, 2017; Zipfel and Oldroyd, 2017). Specialization and adaptation to respective niche has been uncovered by experiments on the competition of the leaf- and root-derived synthetic communities although an extensive taxonomic overlap between leaf and root isolates of *Arabidopsis* has been discovered (Bai et al., 2015).

Phylosymbiosis is a newly formulated concept referring to the process by which the phylogeny of host species parallels the ecological relatedness of corresponding microbial communities (Brooks et al., 2016; Lim and Bordenstein, 2020; O’Brien et al., 2020). This relationship has been detected in a diverse range of taxa and environments, e.g., the gut microbiome of mammals and insects (Kohl et al., 2018), the microbiome of coral reef invertebrates (O’Brien et al., 2020), and the root microbiome of plants (Yeoh et al., 2017). Phylosymbiosis is observed at one moment in time and space, which does not necessarily imply coevolution of hosts and their microbiota, but coevolution may be one mechanism contributing to observations of phylosymbiosis (O’Brien et al., 2020). Ecological and evolutionary mechanisms (selection, dispersal, drift, and diversification) underlying patterns of phylosymbiosis in host-associated microbial communities have been discussed (Kohl, 2020). Host selection or filtering of microbial communities is largely likely to be a dominant contributor to phylosymbiosis, yet, dispersal, drift, diversification, and interactions between these processes may also work (Kohl, 2020).

Bamboo is perennial flowering plant belong to subfamily Bambusoideae of the family Poaceae. It is a kind of the grass plant, but has woody stems. They are widely distributed in every continent except Europe and Antarctica (Li et al., 2006; Clark et al., 2015). Three phylogenetically supported lineages of bamboos have been proposed: temperate woody (Arundinarieae), tropical woody (Bambuseae) and herbaceous (Olyreae) bamboos with their divergence time estimated to be 12.72 million years ago (Mya), 25.86 and 28.26 Mya, respectively (Zhang et al., 2016). Both lineages of woody bamboos are characterized by complex rhizome systems, tree-like habit with highly lignified and usually hollow culms, well-differentiated culm leaves, well-developed aerial branching, foliage leaf blades with outer ligules (Clark et al., 2015). It is worth noting that woody bamboos exhibit extremely long intervals between flowering periods (7–120 years) followed by death of the parent plants (monocarpy) with few seeds produced (Clark et al., 2015; Wysocki et al., 2016). In addition, Moso bamboo (*P. edulis*) can finish their height (10–20 m) and diameter (8–16 cm) growth within 35–40 days with the maximum height growth rate reaching 1–1.5 m within a day (Song et al., 2016; Yen, 2016). Such special biological characteristics made bamboo a perfect candidate for studying the interaction between plants and microbiomes.

A small amount of research concerning bamboos and their associated microbes have been conducted previously, revealing that different bamboo species are colonized by various endophytes (Han et al., 2009; Xu et al., 2014), some of which were growth-promoting bacteria capable of dissolving phosphorus and potassium as well as fixing nitrogen (Yuan et al., 2015b, 2018). However, these researches were largely culture-dependent and restricted to single or few species, likely to underestimate the exact diversity and composition of bamboo endophytes. And no phylosymbiosis research have been reported concerning bamboos and their microbiota. Here, by extensive sampling and using next-generation sequencing, we attempt to answer the following questions:

I. Whether host identity exerts influence on the assemblage of bamboo microbiota.
II. Whether niche differentiation contribute to the variation in root and leaf-associated microbiota?
III. Is there any phylosymbiosis cue between host bamboos and their endophytic partners?

## Materials and Methods

### Sample Collection and Preparation

To investigate whether there is a phylosymbiotic pattern between host bamboos and their endophytes, 15 different bamboo species varying in evolution history, rhizome types, and lifeforms were selected (Table 1). Thirteen woody bamboo species were sampled from Anji Bamboo Expo Park (ABEP: 30°35′19′′N, 119°39′39′′E) in Zhejiang Province, China on October 12th, 2018, and two herbaceous bamboo species, *Mniochloa abersend* and *Olyra latifolia* were sampled from Zhejiang A&F University (ZAFU: 30°15′22′′N, 119°43′44′′E) on October 13th, 2018 (Table 1). Health and fresh leaves and roots (about 10 cm underground) were collected one sample type per species, that is 15 leaf samples and 15 root samples in total. Disposable gloves were changed each time during different sample collection. Samples were kept separately in sterile tubes, stored in liquid nitrogen, and transferred to the lab as soon as possible.

**TABLE 1:** General information of the 15 sampled bamboo species.

Sample ID*	Scientific name	Rhizome types	Lifeforms	Sampling site
BF/FBF/RBF	*Bashania fargesii*	Short-necked pachymorph	Temperate woody	Outdoor, ABEP
BM/FBM/RBM	*Bambusa multiplex*	Leptomorph	Tropical woody	Outdoor, ABEP
CM/FCM/RCM	*Chimonobambusa marmorea*	Leptomorph	Temperate woody	Outdoor, ABEP
DS/FDS/RDS	*Dendrocalamus sinicus*	Short-necked pachymorph	Tropical woody	Greenhouse, ABEP
HT/FHT/RHT	*Hibanobambusa tranguillans*	Leptomorph	Temperate woody	Outdoor, ABEP
IG/FIG/RIG	*Indocalamus guangdongensis*	Leptomorph	Temperate woody	Outdoor, ABEP
MA/FMA/RMA	*Mniochloa abersend*	Short-necked pachymorph	Herbaceous	Greenhouse, ZAFU
OL/FOL/ROL	*Olyra latifolia*	Short-necked pachymorph	Herbaceous	Greenhouse, ZAFU
PH/FPH/RPH	*Phyllostachys heterocycla*	Leptomorph	Temperate woody	Outdoor, ABEP
PP/FPP/RPP	*Pseudostachyum polymorphum*	Leptomorph	Tropical woody	Greenhouse, ABEP
SA/FSA/RSA	*Sasa auricoma*	Leptomorph	Temperate woody	Outdoor, ABEP
SG/FSG/RSG	*Sasaella glabra*	Leptomorph	Temperate woody	Outdoor, ABEP
SK/FSK/RSK	*Shibataea kumasaca*	Leptomorph	Temperate woody	Outdoor, ABEP
ST/FST/RST	*Phyllostachys tootsik*	Leptomorph	Temperate woody	Outdoor, ABEP
TO/FTO/RTO	*Thyrsostachys oliveri*	Short-necked pachymorph	Tropical woody	Greenhouse, ABEP

*^∗^F representing leaf samples and R representing root samples.*

Soil and plant relicts adhering to the surface of root and leaf samples were removed manually in running water. Samples were kept in running water for 1–2 h and were surface-sterilized in 75% ethanol (1 min for leaves and 2 min for roots). Afterward, samples were washed three times with distilled water and sterilized again with 2.5% sodium hypochlorite (5 min for leaves and 10 min for roots) using a vacuum filter pump followed by another 5 times washing with distilled water. Sterility was assessed by placing 100 μl of the last washing water to Luria-Bertani agar (LB) plates for 3–7 days cultivated at 28°C incubator. Samples with no growth on LB plates were used for DNA extraction.

### Sequencing and Phylogenetic Analysis

Total genomic DNA, including the host DNA as well as its endophytes, was extracted using a modified cetyltrimethylammonium bromide (CTAB) method. The V5–V7 region of the bacterial 16S rRNA gene was amplified by degenerated PCR primers 799F and 1193R (Bulgarelli et al., 2012). The PCR reaction was conducted in a 20 μl sample with 10 ng of template DNA, 0.4 μl TransStart Fastpfu DNA Polymerase, 4 μl 5× FastPfu Buffer, 2 μl dNTP (2.5 mM), and 0.8 μl of each primer (5 μM). After an initial denaturation step at 95°C for 3 min, the targeted region was amplified by 27 cycles of 95°C for 30 s, 55°C for 30 s and 72°C for 45 s, followed by a final elongation step of 10 min at 72°C with the 799F and 1392R primers. The second-step primers were 799F-1193R and identical conditions to the first step of the PCR were applied with 15 cycles. The PCR products were analyzed on 2% agarose gel electrophoresis. Amplicon libraries as well as DNA sequencing was conducted using an Illumina MiSeq PE300 platform by Shanghai Majorbio Bio-Pharm Technology Co., Ltd., following the manufacturer’s protocols.

Five plastid intergenic spacers *rbc*L*-psa*I, *rpl*32-*trn*L, *rps*15-*ndh*F, *trn*T-*trn*L, and *ycf*4-*cem*A were amplified (Zhang et al., 2016) and sequenced for phylogenetic analysis (Supplementary Table S1). After alignment, these five plastid DNA sequences were combined, generating a matrix with the length of 3,574 bp. Maximum likelihood (ML) analysis was conducted in MEGA7.0.26 (Kumar et al., 2016) with Tamura 3-parameter and Gamma distribution treated as the best-fit model. The bootstrap values were calculated based upon 1,000 replicates.

### Bioinformatics Analysis on 16S rRNA Gene Profiling

The processing of 16S rRNA sequences was referred to Zheng et al. (2018); Zhang et al. (2019), and Zheng and Gong (2019) using QIIME (Caporaso et al., 2010), USEARCH (Edgar, 2010), and in-house scripts. Briefly, after generation of high-quality reads, unique reads were clustered into OTUs with 97% similarity. Taxonomy of the representative OTUs was classified with the RDP classifier (Wang et al., 2007) based on SILVA 132 database (Quast et al., 2012).

Each sample was rarefied to the lowest sequencing depth 8,337 (in sample RBF) to minimize the potential sampling or sequencing errors, which generating 926 OTUs. The inverse Simpson diversity index (Simpson, 1949), the OTU richness, and the Pielou’s evenness index (Pielou, 1966) were calculated with 999 permutations in R *v*. 3.6.1 (R Core Team, 2019). Normal distribution of the data was checked with the Shapiro–Wilk test and homogeneity of variances were analyzed using the Levene test in R with the ‘car’ package (Fox and Weisberg, 2019). Analysis of Variance was performed to verify whether significant difference of microbial diversity existed among different bamboo species (Rinke et al., 2013). Further, multiple pairwise-comparison between the mean of groups at 95% family-wise confidence level was conducted based on the Tukey Honest Significant Differences test (Tukey HSD).

To compare the composition of identified community members within different bamboo species as well as between leaf and root samples, and to identify main factors driving community structure differentiation, Bray-Curtis dissimilarity matrices was developed with the normalized sequences. Principal coordinates analysis (PCoA) and hierarchical cluster analysis (HCA) of microbial community structure were performed based on the Bray-Curtis dissimilarity matrix by online tool iSanger^1^ with ‘Pearson’ distance measure and the ‘ward’ clustering algorithm. To statistically support the above-mentioned visual clustering results of microbial community composition, permanova and pairwise comparison were conducted using ‘adonis’ and ‘pairwise.adonis’ functions with the ‘bray’ method and 10,000 iterations by ‘vegan’ package in R (Oksanen et al., 2019). By using the multipatt function of the ‘indicspecies’ package in R, indicator species analysis was carried out (Cáceres and Legendre, 2009). Before indicator species calculation, sequences were rarefied as in alpha diversity analysis, and the full genus matrices were retrieved.

To have a comprehensive impression of the microbial community differentiation among different bamboo species, the top 20 genera were treated as core microbiota accounting for 82.25% of total sequences (core root microbiota: 77.63%; core leaf microbiota: 92.46%). We further compared the top 20 leaf and root genera of Arabidopsis (Bai et al., 2015), maize (Niu et al., 2017; Wagner et al., 2020), rice (Zhang et al., 2019; Roman-Reyna et al., 2020) and sugarcane (top 7 genera of leaf microbiota) (Hamonts et al., 2018) with bamboo core microbiota. ANOVA analysis was conducted to check whether the host phylogeny, rhizome type and plant compartment influence the microbial relative abundance. The linear discriminant analysis (LDA) effect size (LEfSe) was calculated to check the difference among samples (Segata et al., 2011). Additionally, Venn diagrams were drawn displaying the overlap of OTUs revealed by different data analysis methods by using BIOINFOGP (Oliveros, 2007). Analysis of differential OTU abundance and taxa was performed using Wilcoxon rank sum test at both OTU level and genus level, and corresponding P values were corrected for multiple test using a false discovery rate (FDR) set at 0.05. Functional prediction and metabolic characteristics of representative OTUs were carried out using PICRUSt (Langille et al., 2013) and FAPROTAX (Louca et al., 2016) with significantly differed catalogs between leaf and root microbiota estimated in STAMP with Welch’s t test corrected by Bonferroni (Parks et al., 2014). Phylogenetic tree of bamboo endophytes was reconstructed from representative OTU sequences from 54 genera with relative abundance higher than 0.2% and visualized by online tool iTOL^2^.

## Results

### Bamboo Leaf and Root Microbiota Do Not Show Significantly Different Alpha Diversity

To explore the variation between leaf and root microbiome, 30 samples from 15 different bamboo species were collected from Anji Bamboo Expo Garden and Zhejiang A&F University (Table 1). After quality control, a total of 6,61,180 high-quality sequences of 16S rRNA gene were recovered from leaf and root samples with the average length of 376.89 bp. Roots contained a little higher 16S rRNA gene sequences than leaf samples (3,43,821 vs. 3,17,359). No Archaeal and Chloroplast sequences were co-amplified during the sequencing process. A small portion of Mitochondrial and chimeric sequences were detected and removed in the downstream analysis. The remaining sequences were clustered into 4,868 operational taxonomic units (OTUs) at a sequence similarity threshold of 97%. Taxonomic annotation was performed with the RDP classifier against the SILVA132 database. To account for differences in sequencing read depth across samples, all samples were rarefied to 8,337 reads per sample based on the minimum read number detected in sample RBF with 926 OTUs detected. Rarefaction analysis revealed that the volume of sequenced reads was sufficient to cover the bacterial diversity within each sample, which suitable for microbiome comparison between samples.

**FIGURE 1:**
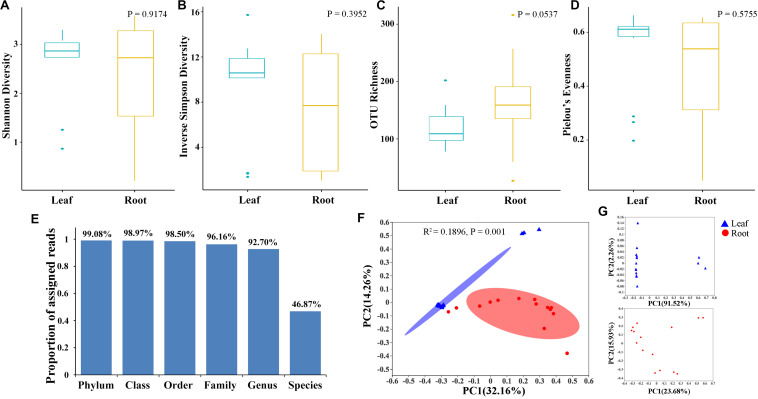
Diversity and composition of bamboo leaf- and root-associated microbiota. The Shannon diversity index **(A)**, Inverse Simpson diversity index **(B)**, OTU richness **(C)**, and Pielou’s evenness **(D)** were calculated with 999 permutations in R *v*. 3.6.1 (R Core Team, 2019). Kruskal–Wallis rank sum test was conducted to test whether there was significant difference of microbial diversity between leaf and root samples with *P*-values presented in the upper right of each graph. Box plots showed the range of estimated values between 25 and 75%, the median, the minimum, and the maximum observed values within each dataset. **(E)** Proportion of total bacterial reads assigned to different taxonomic ranks. **(F)** Principal coordinates analysis (PCoA) of microbial community composition between leaf and root samples at genus level based on the Bray–Curtis dissimilarity matrix performed by online tool iSanger (https://cloud.majorbio.com). The significant difference between leaf and root microbiota was evaluated by ‘adonis’ with 999 iterations. Ellipses cover the data for each compartment. **(G)** Principal coordinates analysis of leaf (up) and root (bottom) microbial community.

The alpha diversity within individual samples was evaluated by the Shannon diversity index, inverse Simpson diversity index, OTU richness, and the Pielou’s evenness (Figures 1A–D). Apart from the OTU richness, where a higher richness value was observed in root (158.40 ± 70.25) than in leaf samples (117 ± 32.65), other three diversity indices exhibited higher values for bamboo leaves. The Shannon diversity indices of the leaf samples (2.58 ± 0.75) were comparable with those of the root ones (2.35 ± 1.19). Kruskal–Wallis rank sum test indicated that endophytic diversity was not significantly different between leaf and root samples as well as among different bamboo species (*P* > 0.05).

To have a comprehensive view of the composition of microbiota in leaf and root samples, OTUs were further assigned to different taxonomic levels by referring to SILVA dataset (Supplementary Table S2). OTUs annotated as unclassified, uncultured and no rank were not discussed in the following analysis as no taxonomic information available with these OTUs. Finally, a total of 294 genera belonging to 171 families, 88 orders, 31 classes and 20 phyla were assigned. Proteobacteria was the predominant phylum compared to the other 19 assigned phyla with its relative abundance account for 63.30% ± 0.27 (±standard deviation) (Leaf: 65.37% ± 0.24; Root: 61.23% ± 0.30). However, the second most abundant phylum, i.e., Bacteroidetes differed between leaf and root samples. Bacteroidetes was abundant in leaf samples (31.63% ± 0.25), whereas Actinobacteria was the second most frequently detected bacterial taxa (28.63% ± 0.27) in roots rather than the Bacteroidetes (6.70% ± 0.07). Proteobacteria was further divided into Gammaproteobacteria, Alphaproteobacteria, and Deltaproteobacteria at the class taxonomic level with Gammaproteobacteria dominant. At lower taxonomic ranks, 92.70% of bacterial reads were successfully annotated at the genus level, however, only 46.87% reads were assigned to species level (Figure 1E). Consequently, downstream analysis was mainly conducted at the genus level and OTU level.

### Beta Diversity Was Remarkably Different Between Bamboo Leaf and Root Microbiota

**FIGURE 2:**
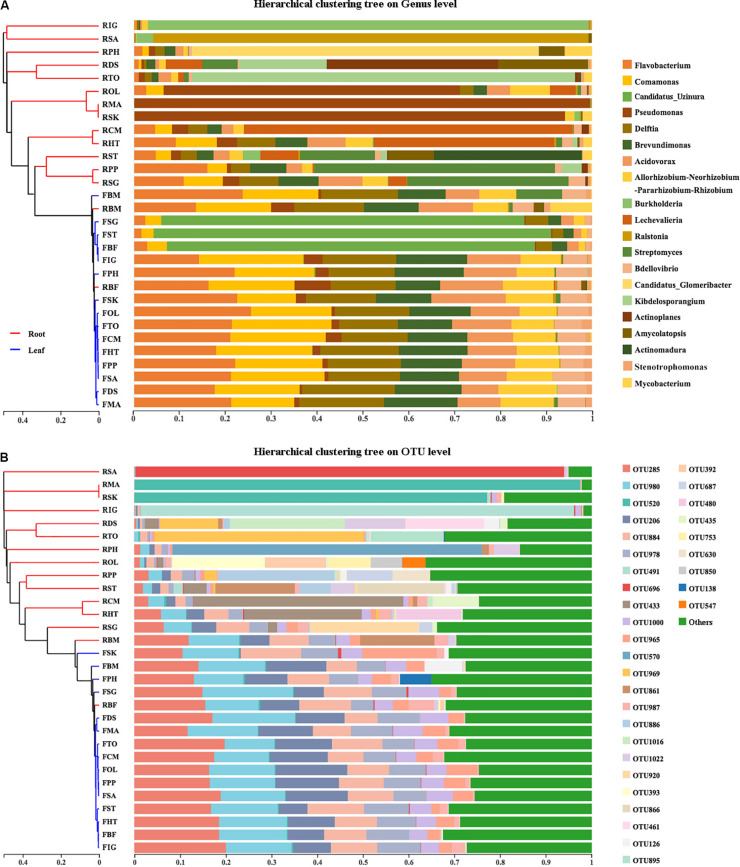
The results of hierarchical cluster analysis (HCA) of bamboo leaf and root microbiota on both Genus **(A)** and OTU **(B)** level. Left was the HCA result showing the microbiota dendrograms derived from 16S rRNA sequences of root and leaf endophytes performed by online tool iSanger (https://cloud.majorbio.com). Pearson distance was applied for HCA. Right was the community structure of root and leaf-associated microbiota with the top 24 genus and top 33 OTUs listed. ‘Others’ was the sum of OTUs with their relative abundance lower than 0.05.

To explore the relative impacts of host compartment, species distribution, and rhizome type on bacterial community composition of 15 bamboo species, PCoA was performed based on the Bray–Curtis distance of all the samples at the genus level (Figure 1F). As a result, host identity did not significantly influence community composition (data not show) whereas plant compartment was proved to be the best factor in explaining microbial variation (ADONIS, *R*^2^ = 0.1896, *P* = 0.001). Leaf microbiota of different bamboo species exhibited similar community composition, except FBF, FSG and FST, however, pronounced variation of microbial composition among root samples were detected. We thus conducted PCoA analysis of leaf and root microbiota separately (Figure 1G). From the PCoA result of leaf microbiota (Figure 1G: Top), two groups could be clustered. One included FBF, FSG, and FST, and the other included the remaining 12 samples. Similar microbial composition was also found in bar plots among all the 15 leaf samples both at genus (Figure 2A) and OTU level (Figure 2B). However, no such homogeneity was detected in root microbiota. Root sample RBF and RBM showed some similar bacterial composition with the leaf samples (Figure 2A, right). Therefore, we treated the 15 different bamboo species as replicates for leaf and root samples, respectively (Xu et al., 2018).

Hierarchical cluster analysis (HCA) of the microbial community structure was performed to explore any correlation between host plants and their endophytes. Two major groups were identified from hierarchical cluster analysis at both genus level (Figure 2A, left) and OTU level (Figure 2B, left). All the leaf samples formed a monophyletic group with two root samples RBF and RBM insert which is in accordance with community composition analysis (Figure 2, right).

### Testing the Phylosymbiotic Relationship Between Host Bamboos and Microbiota

**FIGURE 3:**
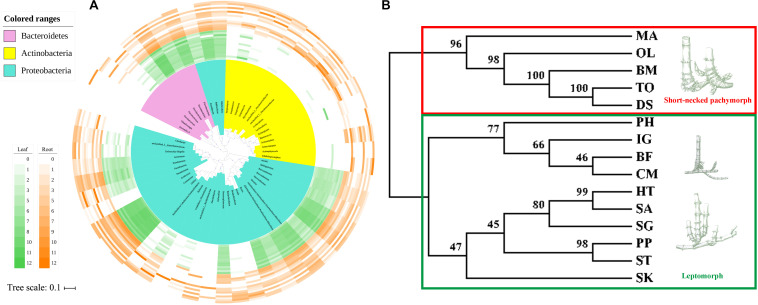
The taxonomic topology of bamboo leaf and root microbiota and their hosts’ phylogeny. **(A)** The microbiota dendrogram was constructed based on OTUs with relative abundance higher than 0.2% using the maximum-likelihood method. The inner colored ranges identify phyla within the tree. The outer colored bars represent the relative abundance of each OTU in leaf and root compartments. The figure was visualized by online tool iTOL (http://itol.embl.de). **(B)** The host’s maximum-likelihood phylogeny was deduced based on 5-region cpDNA dataset (*rbc*L*-psa*I, *rpl*32-*trn*L, *rps*15-*ndh*F, *trn*T-*trn*L, and *ycf*4-*cem*A) of the 15 sampled bamboo species using the maximum-likelihood method in MEGA7.0.26. Maximum likelihood bootstrap values were present above the branches. The images of different rhizome root types were displayed along the right side of the ML tree according to the dendrogram cluster.

The taxonomic dendrogram of bamboo leaf and root microbiota at genus-level with their relative abundance higher than 0.2% was generated based on maximum-likelihood method (Figure 3A). The whole microbiota was divided into three major clades. Sequences from phylum Bacteroidetes formed one monophyletic clade. Sequences from Actinobacteria formed another clade and became sister group with two Proteobacteria taxon, Peredibacter and Bdellovibrio. The remaining Proteobacteria grouped together as a single cluster. Bacteroidetes derived from leaf and root of 15 different bamboo species showed almost no niche specialization as they share similar relative abundance among different genera. Same trend was also seen in Proteobacteria, with *Bradyrhizobium* and *Steroidobacter* as exception. *Bradyrhizobium*, *Steroidobacter*, as well as species from Actinobacteria were exclusively enriched in roots.

The maximum-likelihood phylogenetic tree based on the concatenated plastid matrix was constructed to investigate whether there is any phylosymbiosis cue between host bamboos and their microbiota (Figure 3B). Two groups were clearly separated according to the dendrogram structure. One constituted species characterized by pachymorph with short necks (which was supposed to be an ancient trait), including two herbaceous bamboos (MA and OL), and three woody bamboos (BM, TO, and DS). The remaining 10 bamboo species clustered together as leptomorph (more derived trait). These results were consistent with the bamboo phylogeny inferred by Zhang et al. (2016) based on six cpDNA genes. However, it was inconsistent with the hierarchical clustering trees constructed by microbial data derived from bamboo leaves and roots. The result of Mantel test verified that the correlation between microbiota compositional similarity and bamboos’ phylogenetic relatedness is insignificant (Mantel statistic *r* = −0.1809, *P* = 0.966), indicating that no phylosymbiosis was observed between bamboos and their associated microbiota.

### Identification of Differential Bacteria Between Root and Leaf Microbiota

**FIGURE 4:**
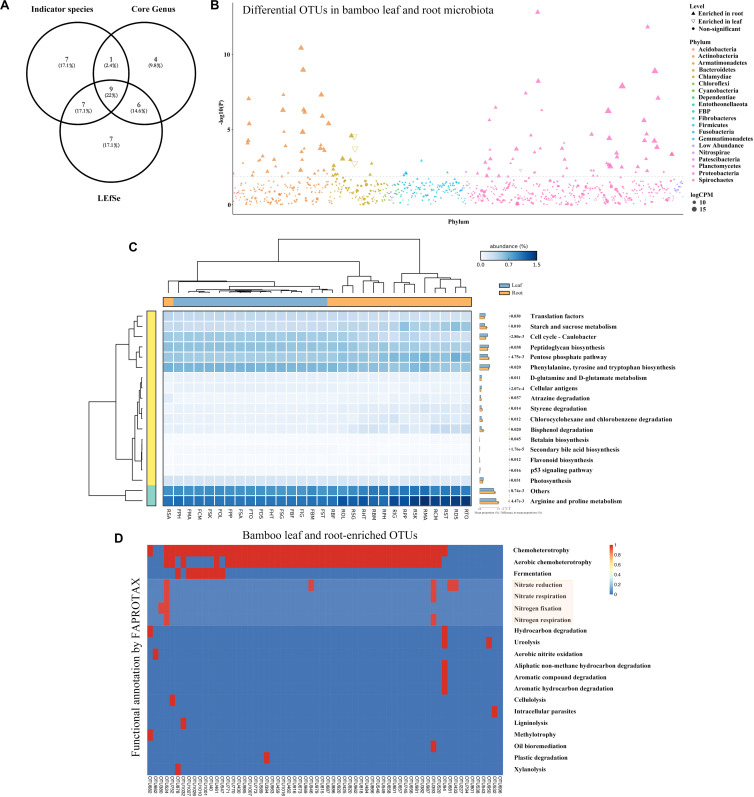
The comparison and functional prediction of differential bacteria between leaf and root microbiome. **(A)** The comparison of bamboo genus generated by indicator species, core genus and LEfSe by Venn diagram. **(B)** Manhattan plot showing OTUs enriched in leaf and root samples. Each dot or triangle represented a single OTU. OTUs enriched in root or leaf are represented by filled or empty triangles, respectively (FDR adjusted *P* < 0.05, Wilcoxon rank sum test). OTUs are arranged in taxonomic order and colored according to the phylum. CPM, counts per million. **(C)** Functional analysis of bamboo-associated microbiome predicted by PICRUSt with their statistical significance estimated in STAMP. From left to middle and right were the phylogeny of annotated functions, heatmap showing the average percentage of annotated proteins of each cluster belonging to KEGG functional category 3, and the mean proportions of significantly differed proteins between leaf and root microbiome, respectively. **(D)** Metabolic and ecological functions of bamboo associated OTUs annotated by FAPROTAX. Each row represents an OTU and the presence of functions is shown in red. Functions related to nitrogen fixation were covered by light red.

To find the potential microbes that may contribute to the differentiation of microbiota between roots and leaves, core microbes, indicator species and LEfSe were analyzed at genus level (Figure 4A and Table 2). The indicator species analysis identified 24 genera in all samples. The top 20 core microbes accounted for 82.25% of the total reads, with leaf and root microbiota occupying 92.46 and 77.63% reads, respectively. Specifically, *Candidatus uzinura* (Leaf: 15.96% ± 0.32 vs. Root: 0.00%), *Flavobacterium* (Leaf: 14.92% ± 0.07 vs. Root: 3.96% ± 0.04), and *Comamonas* (Leaf: 13.58% ± 0.06 vs. Root: 3.41% ± 0.05) were more abundant in leaf compartment than in root; whereas *Pseudomonas* (Leaf: 1.23% ± 0.01 vs. Root: 14.65% ± 0.29) was dominant in root samples. Leaf microbiota contained a little higher number of significantly differed genera as compared to the root (LEfSe: 16 in leaves vs. 13 in roots). *Acidovorax*, *Allorhizobium_Neorhizobium_Pararhizobium_Rhizobium*, *Bdellovibrio*, *Brevundimonas*, *Candidatus uzinura*, *Comamonas*, *Delftia*, *Flavobacterium*, and *Stenotrophomonas* were the 9 common genera revealed by indicator species, LEfSe and top 20 genera analysis (Figure 4A and Table 2). Among the detected genera, plant growth promoting bacterial, *Bradyrhizobium*, *Citrobacter*, *Mycobacterium*, and *Streptomyces* were exclusively enriched in root and *Aeromonas*, *Allorhizobium_Neorhizobium_Pararhizobium_Rhizobium*, *Brevundimonas*, *Flavobacterium*, and *Stenotrophomonas* were exclusively enriched in leaf samples. Their potential functions will be discussed in the following section.

**TABLE 2:** Results of indicator genus, core genus and the linear discriminant analysis (LDA) effect size (LEfSe) analysis.

Indicator genus	Leaf^ a^	Root	Indicator value	*P*-value^*b*^	Core genus	Leaf	Root	LEfSe	Group	LDA score (log10)	*P*-value
***Candidatus_Uzinura***	15.96% ± 0.32	0.00%	1.000	0.001***	***Flavobacterium***	14.92% ± 0.07	3.96% ± 0.04	***Acidovorax***	Leaf	4.343	0.008**
*Pseudomonas*	1.23% ± 0.01	14.65% ± 0.28	0.985	0.001***	***Comamonas***	13.58% ± 0.06	3.41% ± 0.05	*Aeromonas*	Leaf	3.719	0.000***
*Labrys*	0.00%	0.01% ± 0.00	0.996	0.002**	***Candidatus_Uzinura***	15.96% ± 0.32	0.00%	***Allorhizobium***	Leaf	4.361	0.001***
***Bdellovibrio***^*c*^	4.33% ± 0.02	1.12% ± 0.01	0.863	0.003**	***Delftia***	11.93% ± 0.05	3.19% ± 0.04	***Bdellovibrio***	Leaf	4.205	0.000***
***Brevundimonas***	9.99% ± 0.04	2.92% ± 0.03	0.853	0.003**	***Brevundimonas***	9.99% ± 0.04	2.92% ± 0.03	*Bosea*	Leaf	3.629	0.007**
***Flavobacterium***	14.92% ± 0.07	3.96% ± 0.04	0.912	0.003**	***Acidovorax***	8% ± 0.03	3.03% ± 0.03	***Brevundimonas***	Leaf	4.537	0.000***
*Herminiimonas*	0.00%	0.08% ± 0.00	1.000	0.003**	***Allorhizobium***	7.01% ± 0.03	2.44% ± 0.02	***Comamonas***	Leaf	4.709	0.000***
***Acidovorax***	8.00% ± 0.03	3.03% ± 0.03	0.857	0.004**	***Bdellovibrio***	4.33% ± 0.02	1.12% ± 0.01	***Delftia***	Leaf	4.634	0.000***
*Acidothermus*	0.00%	0.01% ± 0.00	0.985	0.005**	*Pseudomonas*	1.23% ± 0.01	14.65% ± 0.29	*Devosia*	Leaf	3.915	0.001***
***Allorhizobium***^*d*^	7.01% ± 0.01	2.44% ± 0.02	0.824	0.005**	***Stenotrophomonas***	0.95% ± 0.00	0.32% ± 0.00	***Flavobacterium***	Leaf	4.723	0.000***
*Bosea*	0.60% ± 0.00	0.24% ± 0.00	0.791	0.007**	*Streptomyces*	0.73% ± 0.02	5.43% ± 0.11	*Peredibacter*	Leaf	3.818	0.002**
*Devosia*	0.33% ± 0.00	0.15% ± 0.00	0.796	0.007**	*Ralstonia*	0.12% ± 0.00	6.38% ± 0.23	*Shinella*	Leaf	3.824	0.001***
***Delftia***	11.93% ± 0.04	3.19% ± 0.04	0.843	0.008**	*Mycobacterium*	0.05% ± 0.00	1.2% ± 0.02	*Sphingobacterium*	Leaf	3.722	0.002**
***Stenotrophomonas***	0.95% ± 0.00	0.32% ± 0.00	0.805	0.008**	*Burkholderia*	0.02% ± 0.00	6.84% ± 0.24	*Sphingomonas*	Leaf	3.877	0.004**
***Comamonas***	13.58% ± 0.06	3.41% ± 0.04	0.844	0.009**	*Lechevalieria*	0.01% ± 0.00	6.81% ± 0.15	***Stenotrophomonas***	Leaf	3.664	0.003**
*Shinella*	0.46% ± 0.00	0.11% ± 0.00	0.831	0.009**	*Kibdelosporangium*	0.02%	4.27% ± 0.12	***Candidatus_Uzinura***	Leaf	4.946	0.004**
*Gordonia*	0.01% ± 0.00	0.00%	0.918	0.012*	*Actinomadura*	0.00%	1.3% ± 0.05	*Acidibacter*	Root	3.546	0.000***
*Bradyrhizobium*	0.00%	0.96% ± 0.02	0.950	0.017*	*Actinoplanes*	0.00%	1.92% ± 0.06	*Actinomadura*	Root	4.114	0.008**
*Xanthobacter*	0.00%	0.01% ± 0.00	0.801	0.022*	*Amycolatopsis*	0.00%	1.85% ± 0.04	*Actinoplanes*	Root	4.101	0.002**
*Rhodoplanes*	0.01% ± 0.00	0.01% ± 0.00	0.892	0.031*	*Candidatus Glomeribacter*	0.00%	4.59% ± 0.17%	*Amycolatopsis*	Root	4.096	0.000***
*Shewanella*	0.14% ± 0.00	0.05% ± 0.00	0.800	0.035*				*Bradyrhizobium*	Root	3.859	0.000***
*Sphingomonas*	0.65% ± 0.00	0.29% ± 0.00	0.756	0.041*				*Citrobacter*	Root	4.045	0.020*
*Aeromonas*	0.44% ± 0.00	0.10% ± 0.00	0.792	0.044*				*Kibdelosporangium*	Root	4.504	0.049*
*Sphingobacterium*	0.22% ± 0.00	0.04% ± 0.00	0.862	0.050*				*Lechevalieria*	Root	4.683	0.000***
								*Mycobacterium*	Root	3.861	0.000***
								*Ohtaekwangia*	Root	3.756	0.035*
								*Phenylobacterium*	Root	3.771	0.015*
								*Steroidobacter*	Root	3.719	0.003**
								*Streptomycs*	Root	4.464	0.034*

*^a^The average relative abundance of leaf and root microbiota ± standard deviation. ^b^Significance levels: ^∗^0.01 < p ≤ 0.05, ^∗∗^0.001 < p ≤ 0.01, ^∗∗∗^p ≤ 0.001. ^c^Genus in bold font indicates the 9 common genera shared among indicator species, core microbes, and the linear discriminant analysis (LDA) effect size (LEfSe) analysis. ^d^Allorhizobium is short for Allorhizobium_Neorhizobium_Pararhizobium_Rhizobium.*

To capture any enriched/depleted OTUs in different compartments, the difference of microbiota between leaf and root samples at OTU level was examined by conducting Manhattan plot (Figure 4B). As expected, 112 OTUs were specifically enriched in root and 4 were enriched in leaf samples (FDR adjusted *P* < 0.05, Wilcoxon rank sum test). Among the enriched OTUs, 47.86% belonged to Proteobacteria, and 34.19% belonged to Actinobacteria (Supplementary Table S3).

**FIGURE 5:**
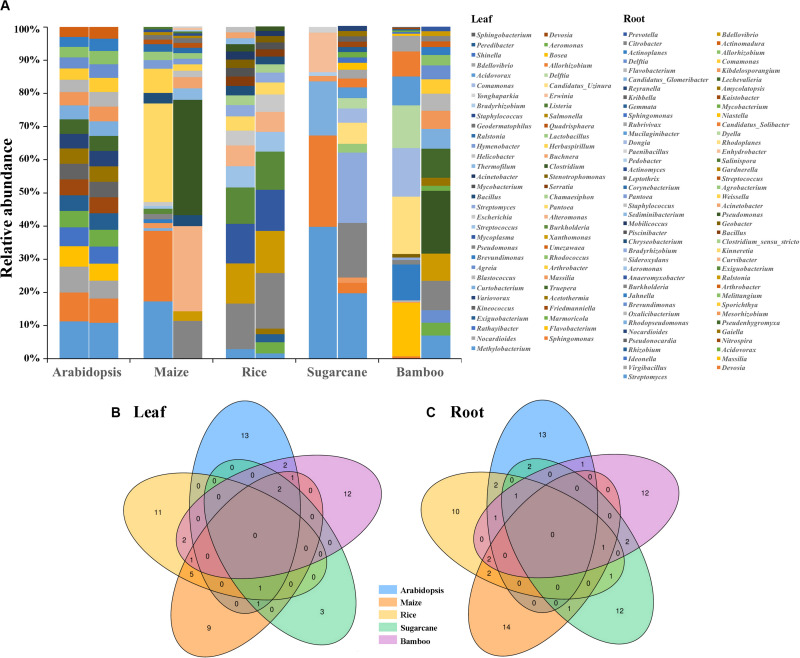
The comparison of core microbiota among Arabidopsis (Bai et al., 2015), maize (Niu et al., 2017; Wagner et al., 2020), rice (Zhang et al., 2019; Roman-Reyna et al., 2020), sugarcane (Hamonts et al., 2018), and bamboos. **(A)** The composition of leaf and root core microbiota (top 20 genera, with top 7 genera of leaf microbiota for sugarcane) among different plants. Left bar plot was the leaf microbiota and right was the root microbiota. Venn diagrams indicated the number of common and species-specific leaf **(B)** and root **(C)** core genera among Arabidopsis, maize, rice, sugarcane, and bamboos.

The comparison of core microbes among Arabidopsis, maize, rice, sugarcane, and bamboos revealed that different plants exhibited different core microbiota (Figure 5A). None of the core microbiota were shared among these five plant taxa with most of them species-specific (Figures 5B,C). In addition, compartment-specific was uncovered for some species. The composition of Arabidopsis leaf and root core microbiota were somewhat similar with their relative abundance varying slightly, the same is true of rice. However, for maize, sugarcane and bamboos, different compartment (leaf vs. root) have different microbial composition. For example, the most abundant genus of bamboo leaf microbiota was *Candidatus_Uzinura* (15.96%), followed by *Flavobacterium* (14.92%) and *Comamonas* (13.58%). However, the most abundant genus for bamboo root microbiota was *Pseudomonas* (14.76%), *Burkholderia* (6.84%), and *Lechevalieria* (6.81%). Additionally, the top 3 genera of root microbiota were *Pseudomonas* (23.01%), *Curvibacter* (17.03%), *Burkholderia* (7.52%) for maize, and *Bradyrhizobium* (2.07%), *Streptomyces* (1.92%), *Burkholderia* (1.61%) for sugarcane, respectively. The top 3 genera for maize and sugarcane leaf microbiota were *Pantoea* (28.95%), *Sphingomonas* (20.7%), *Methylobacterium* (16.85%), and *Methylobacterium* (12.58%), *Sphingomonas* (8.7%), and *Curtobacterium* (5.18%), respectively. Accordingly, we propose that the core microbiota of different plants is somewhat species-specific, which may result from the evolutionary conservation of certain microbes, coupled with the influence of environmental factors (Yeoh et al., 2017).

### Function Prediction and Metabolic Characteristics

In this study, analogous KEGG orthology pathways were found between root and leaf microbiota, indicating that these two niches had functional overlapped microbiota (Figure 4C). Most of the annotated OTUs were related with ‘metabolism’ at the level 1 category, followed by ‘environmental information processing’ and ‘genetic information processing.’ At level 2 category, 3 of the 41 predicted metabolism pathways differed significantly between root and leaf samples (Welch’s *t*-test corrected by Bonferroni, *P* < 0.05; Supplementary Table S4). Function of ‘metabolism’ was specially enriched in root microbiome. The other 2 functional traits, ‘environmental adaptation’ and ‘cell growth and death,’ were elevated in leaf microbiota. At level 3 category, 19 functional traits were remarkably different between leaf and root microbiota (Welch’s *t*-test corrected by Bonferroni, *P* < 0.05; Figure 4C and Supplementary Table S4). Most of them were related to amino acid metabolism, such as ‘arginine and proline metabolism,’ ‘phenylalanine, tyrosine and tryptophan biosynthesis.’ Some were associated with carbohydrate metabolism, including ‘pentose phosphate pathway’ and ‘starch and sucrose metabolism.’ Some were ‘cellular antigens’ for signaling molecules and interaction. Others were related to cell growth and death, like ‘p53 signaling pathway.’ Interestingly, most of the metabolism-related functions were uniquely enriched in root microbiota.

**FIGURE 6:**
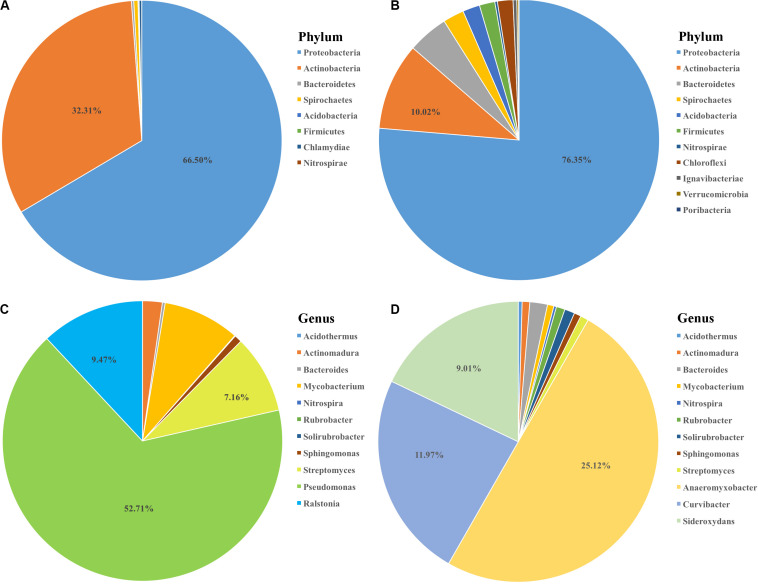
The comparison of nitrogen metabolism-associated microbiota between bamboo and rice *indica* (Zhang et al., 2019). The composition of potential nitrogen metabolism related microbiota at phylum level for bamboo **(A)** and *indica* **(B)** with the proportion of most abundant two phyla list. The 9 common genera shared between bamboo **(C)** and *indica* **(D)** coupled with the proportion of three most dominant genera list.

To further investigate whether if any detected OTUs had functions related to nitrogen cycle, OTU functions were additionally annotated by FAPROTAX database (Louca et al., 2016). 64 OTUs were found to be related to 20 functional traits (Figure 4D). Chemoheterotrophy was the most dominant item with 52 out of 64 OTUs included, followed by aerobic chemoheterotrophy. Four nitrogen metabolism related functions, including nitrate reduction, nitrate respiration, nitrogen fixation and nitrogen respiration, were annotated. Notably, one root enriched OTU (OTU524, annotated as *Azospira*), was predictively included in all the four nitrogen metabolism pathways which were likely contributing to the host’s nitrogen level. We then compared the 64 bamboo OTUs with 141 *indica*-enriched OTUs which were reported to be related with nitrogen metabolism in rice (Zhang et al., 2019). Seven phyla and 9 genera were found to be shared between *indica* and bamboo, but their relative abundance varied (Figure 6). For *indica* enriched OTUs, 76.35% were comprised by Proteobacteria, 10.02% by Actinobacteria. However, for bamboos, Proteobacteria accounted for 66.50% of total OTUs and Actinobacteria took up 32.31%. At the genus level, 141 *indica* enriched OTUs were annotated to 76 genera, and bamboo were annotated to 41 genera, among which 9 were shared between them. However, the 9 shared genera only represented 4.17 and 16.97% of the total OTUs for rice and bamboo, respectively. The most abundant genus of *indica* was *Anaeromyxobacter* (25.12%), followed by *Curvibacter* (11.97%) and *Sideroxylon* (9.01%). The most dominant genus for bamboo was *Pseudomonas* (52.71%), followed by *Ralstonia* (9.47%), and *Streptomyces* (7.16%).

## Discussion

### Structural Variation and Niche Differentiation Between Bamboo Root and Leaf Microbiota

Different plant compartments, e.g., leaves, roots, seeds, and flowers, represent unique ecological niches and host a niche-specific core microbial taxon (Muller et al., 2016; Hassani et al., 2018). Research based on poplar (Beckers et al., 2017) and cycad (Zheng and Gong, 2019) verified specialization of microbiota to their respective niches. Here, resembled leaf bacterial diversity and community composition among different bamboo species were detected, yet the root microbiota were highly variable. Structural variability and niche differentiation were further uncovered in the root and leaf microbiota, especially for species from Actinobacteria. The comparison of bamboo microbiota with Arabidopsis, maize, rice, and sugarcane also revealed the species-specific of core microbes with no top genus shared among them. Additionally, the core leaf and root microbiota of maize and sugarcane were different, which accorded closely with our findings. The well-documented plant growth promoting bacterial, *Bradyrhizobium*, *Citrobacter*, *Mycobacterium*, and *Streptomyces* were exclusively enriched in root microbiota, while *Aeromonas*, *Allorhizobium_Neorhizobium_Pararhizobium_Rhizobium*, *Brevundimonas*, *Flavobacterium*, and *Stenotrophomonas* were abundant in leaf samples. Notably, no root-specific endophyte was shared by indicator species, LEfSe and top 20 genera analyses.

Fundamental structure and physiological differences need to be considered when comparing root and leaf microbiota (Singh and Dubey, 2018). On one hand, the studied bamboo species have distinct rhizome types (Clark et al., 2015). Species BM, DS, MA, OL, and TO were featured by short-necked pachymorph, while the remaining nine bamboo species were leptomorph, including monopodial and amphipodial (Zhang et al., 2020). Alternatively, the leaf structure of different bamboo species was quite similar. Different tissue structures selected different kind of microorganisms as their endophytic partners (Zheng and Gong, 2019). On the other hand, structural variation resulted in physiological difference. Roots released large amount of metabolic products into the rhizosphere space to attract as much microbes as possible to colonize the rhizosphere where they could select suitable ones to establish symbiotic relationship (Leach et al., 2017; Zhalnina et al., 2018; Trivedi et al., 2020). The leaf cuticle and cell wall molecules in principle provided organic matter for the adhesion of epiphytic bacteria, however, majority of soluble organic compounds were kept in the interior tissues of leaves, with few available for the phyllosphere microorganisms (Lindow and Brandl, 2003; Leveau, 2006). More importantly, the special cuticle and crystalline plate-like waxes covering bamboo leaves served as physical barrier protecting plants against microorganism invasion (Yeats and Rose, 2013; Serrano et al., 2017; Sharma et al., 2018). Therefore, instead of a gradual substrate-driven community shift of the soil microbiome initiated at a distance from the root in the rhizosphere, the selection of phyllospheric microbial communities appeared to take place solely at the immediate leaf surface, which strongly influence the assemblage of endophytic microbiomes (Bulgarelli et al., 2013). In summary, the structural variations and substrate-driven selection regulated by the differences in the abundance of organic substrate on leaf and root surfaces at least partly drive the differentiation of distinctive microbial assemblages.

### No Phylosymbiosis Between Bamboos and Their Associated Microbiota

Given the highly resembled leaf microbiota among different bamboo species regardless of their distributions (outdoor vs. greenhouse), lifeforms (woody vs. herbaceous) and rhizome types (pachymorph vs. leptomorph), we speculated the possibility of some phylosymbiotic pattern existed between the host plants and their associated microbiota. Because prokaryote-eukaryote interactions are primordial, evolutionary history of eukaryotic host can be a significant factor shaping the composition and structure of associated microbial consortia (Bouffaud et al., 2014; Yeoh et al., 2017). However, we failed to detect any phylosymbiosis evidence between the sampled 15 bamboo species and their microbiota by comparing the phylogeny congruence based on both host’s cpDNA matrix and endophytic 16S rRNA sequences (Mantel statistic *r* = −0.1809, *P* = 0.966). Similar result was detected between skin microbiome and host phylogeny in the case of carnivores (Ross et al., 2018).

O’Brien et al. (2020) proposed three main reasons why phylosymbiosis does not occur. First, host genotype exerts strong effects on microbiota composition that are independent of host phylogeny. Second, host physiology can structure the microbiome (Amato et al., 2019a), which may not always be consistent with host phylogeny (Amato et al., 2019b). Finally, environment conditions may obscure phylosymbiotic signals (Brooks et al., 2016). In addition, eco-evolutionary processes (selection, dispersal, drift, and diversification) (Cordovez et al., 2019; Kohl, 2020), and priority effect (Carlström et al., 2019) may also underlie the phylosymbiosis apart from host selection.

In this study, we found that host identity and their lifeforms didn’t significantly influence the root and leaf microbiota. Patterns of phylosymbiosis may be dependent on a certain host taxonomic level (i.e., family), where bamboo identity effects are reduced and their physiological traits and phylogeny are congruent (O’Brien et al., 2020). The long evolutionary history of bamboos (28.26 Mya) (Zhang et al., 2016), as well as the extremely long intervals between flowering periods (7–120 years) (Kelchner and Bamboo, 2013) would have contributed to phylosymbiosis between host bamboos and microbiota. However, these phylosymbiotic signals may be obscured by the external environmental conditions. Most samples were collected from the same site (ABEP), with MA and OL sampled from ZAFU. These two sites were 37.5 km apart and their climate conditions and environment surroundings quite resemble [both are subtropical monsoon climate with four distinct seasons, mean annual precipitation is 1,485 mm (ABEP) vs. 1,420 mm (ZAFU), and the mean annual temperature is 15°C (ABEP) vs. 15.6°C (ZAFU)] (Song et al., 2016), giving rise to the concordance of leaf microbiota from different bamboo genus. It is also possible that priority effect may play a role, which means that the first microbial colonizers of bamboo organs altered the inner physiology environment by activating the host immune system or though the production of bioactive compounds that inhibit the growth of other microbial species (Kohl, 2020). Nevertheless, more relevant experiments are needed to verify the detailed mechanisms underlying the relationship between bamboos and microbiota.

### Function Overlap of Bamboo Leaf and Root Microbiota

Function overlap between root and leaf microbiota from different bamboo species was detected in current study based on 16S rRNA gene functional prediction. Almost each annotated KEGG functional category was present in leaf and root microbiota with their relative abundance varying. Indeed, similar results have been found in *Arabidopsis* (Bai et al., 2015) and rice (Acosta et al., 2020). Comparison of the culture-dependent diversity of *Arabidopsis* with OTUs detected on leaves and roots of naturally grown plants revealed high functional conservation, even when samples originated from different continents (Bai et al., 2015). Moreover, the similarity of leaf microbiomes found between aquatic duckweed and terrestrial rice and *Arabidopsis* suggests a conserved structuring effect by leaf tissue on plant microbiota over a large evolutionary distance of 100 Myr (Acosta et al., 2020).

Among the significantly differed KEGG categories between root and leaf microbiota, ‘amino acid metabolism,’ ‘cell growth and death,’ ‘glycan biosynthesis and metabolism,’ were mainly present in leaf microbiome, notably phenylalanine, tyrosine and tryptophan biosynthesis, as well as peptidoglycan biosynthesis. Alternatively, ‘carbohydrate metabolism’ and ‘xenobiotics biodegradation and metabolism’ were dominant in root microbiome. Previous research has revealed that the concentration of major metabolites was different between leaf and root. For example, higher level of amino acids and sugars content was detected in roots than in leaves (Dong et al., 2016). The relative lower level of nutrient contents in plant leaves may induce the endophytic bacterial to synthesize amino acids and glycan by themselves, promoting the expression of genes relevant for amino acid metabolism as well as glycan biosynthesis and metabolism. The prevalence of metabolic pathway corresponding to ‘xenobiotics biodegradation and metabolism’ in bamboo roots suggested that they may specially recruit microorganisms capable of degrading pesticides, such as atrazine, and other environmental contaminants that widespread in the soil of the targeted regions where management practices were applied although not frequently. Many soil dwelling Actinobacterial bacteria can degrade a wide range of stable xenobiotics like bisphenol, chlorocyclohexane and chlorobenzene, and styrene (Solyanikova and Golovleva, 2015). The bamboo microbiota may also utilize aromatic compounds from host roots as growth substrates.

In our study, Actinobacteria was specially preferred in root niche which is consistent with Fitzpatrick et al.’s (2018) finding that Actinobacteria were pronouncedly enriched in endosphere of angiosperms under drought condition. A large overlap of metabolic functional capabilities between leaf and root-associated bacteria with few significant differences at the level of individual functional categories were uncovered. Terrestrial plants may recruit Actinobacteria into their root-associated bacterial communities to facilitate adaptation to stresses commonly encountered in terrestrial environments, such as drought (Acosta et al., 2020). Furthermore, *Streptomycs* from Actinobacteria were among the top 20 genera and significantly enriched in root samples as compared to leaf counterparts. *Streptomyces* exhibit traits of potential benefit to host plants, including the production of antimicrobial compounds, thick-walled spores resilient to environmental perturbation, and inducible exploratory behavior (Jones et al., 2017), all of which may increase colonization rates of plant tissue under stressful environments. Strains from *Streptomyces* constantly found to be capable of indole-3-acetic acid (Jones et al.) production, nitrogen fixation, phosphate solubilization, and siderophore production. They can also biodegrade xenobiotics effectively and inhibit plant pathogens, protecting host plants from diseases (Singh and Dubey, 2018).

An unexpected finding in this study is that *C. uzinura* occupied an overwhelming proportion in leaf microbiota (15.96% ± 0.32) than roots’ (0.00%). This genus was an endosymbiont of armored scales, residing within a specialized pentaploid bacteriocytes that dispersed throughout the body cavity (Sabree et al., 2013). The detection of *C. uzinura* was probably either from sample preparation or from sequencing process although relevant testing experiment need to be conducted.

The identification of other plant growth promotion bacteria may play roles in plant growth and adaptation. By comparing the potential nitrogen metabolism-associated microbes of bamboos with that of *indica*-enriched OTUs, 7 phyla and 9 genera were specifically shared among them, indicating that nitrogen metabolism-related microbes may exist in bamboo microbiota which may contribute to bamboo’s fitness. In addition, *Azospira* is a kind of nitrate (NO_3_^–^) reducing Fe (II) oxidizers engaged in biogeochemical cycles of carbon and nitrogen (Li et al., 2016). *Bradyrhizobium* is well-known as root-nodule bacteria of both legumes and non-legumes plants and supply their hosts with biologically fixed nitrogen (Yeoh et al., 2017). Members of the genus *Sphingomonas* suppressed the growth of foliar pathogen *Pseudomonas syringae* on *A. thaliana* under laboratory conditions and protected the plant from disease (Bulgarelli et al., 2015). A *Pseudomonas mediterranea* strain S58 isolated from tobacco rhizosphere proved to be versatile plant growth-promoting agent with multiple beneficial traits for plants, such as solubilizing organic phosphate, producing siderophore, protease, ammonia, and indole-3-acetic acid (Gu et al., 2020).

## Conclusion

The leaf and root compartments host an assembly of taxonomically diverse bacteria, benefiting the growth and performance of the host plants by enhancing nutrient supply and tolerance to stress. We conducted a comprehensive study of bamboo microbiota derived from 15 different genera, covering different rhizome types, lifeforms, and compartments. Structure variation and niche specialization were detected between leaf and root microbiota of 15 bamboo species. Community structure of leaf microbiota highly resembled in contrast to root microbiota regardless of where the bamboo plants grew and which lifeform they led. However, no phylosymbiosis pattern was observed between bamboos and microbiota. A large overlap of metabolic functional capabilities between leaf and root-associated bacteria with few significant differences at the level of individual functional categories were uncovered. Therefore, we propose that environmental conditions, structural variations and fundamental physiological differences between leaf and root compartments conjunctly contributed to the divergence of bamboo microbiota. Functional overlap between leaf and root microbiota represented the evolutionary conservation of certain core microbes, such as *Citrobacter*, *Pseudomonas*, and *Streptomyces*, which may contribute to the host growth and fitness. Our study offers a comprehensive understanding of bamboo-microbe relationships and provides a list of bacterial lineages for investigation into their specific plant–microbe interaction knowledge of which could be used to enhance agricultural and forest productivity.

## Data Availability Statement

The raw 16S rRNA gene amplicon sequences supporting for this study have been deposited in the NCBI Sequence Read Archive (SRA) database under the BioProject accession number PRJNA630384.

## Author Contributions

YZ and XL designed the research. YZ conducted the experiments, analyzed the data, and wrote the manuscript. XL revised the manuscript. Both authors contributed to the article and approved the submitted version.

## Conflict of Interest

The authors declare that the research was conducted in the absence of any commercial or financial relationships that could be construed as a potential conflict of interest.

## Footnotes

1 https://cloud.majorbio.com

2 http://itol.embl.de

## Supplementary Material

TABLE S1 Primer sequences and polymerase chain reaction (PCR) protocols used for the plastid DNA fragments of this study.

TABLE S2 OTUs generated in current study and their taxonomic assignment results.

TABLE S3 OTUs specifically enriched/depleted in root and leaf compartments of bamboos revealed by Manhattan plot.

TABLE S4 Significantly different pathways at KEGG functional category 2 and 3 level between leaf and root microbiomes with statistical significance estimated in STAMP.
